# Fruit and Leaf Sensing for Continuous Detection of Nectarine Water Status

**DOI:** 10.3389/fpls.2019.00805

**Published:** 2019-07-03

**Authors:** Alessio Scalisi, Mark Glenn O’Connell, Dario Stefanelli, Riccardo Lo Bianco

**Affiliations:** ^1^Department of Agricultural, Food and Forest Sciences (SAAF), University of Palermo, Palermo, Italy; ^2^Department of Jobs, Precincts and Regions, Agriculture Victoria, Tatura, VIC, Australia

**Keywords:** drought, fruit growth, irrigation, *Prunus persica* (L.) Batsch, turgor pressure, water potential

## Abstract

Continuous assessment of plant water status indicators provides the most precise information for irrigation management and automation, as plants represent an interface between soil and atmosphere. This study investigated the relationship of plant water status to continuous fruit diameter (FD) and inverse leaf turgor pressure rates (*p*_p_) in nectarine trees [*Prunus persica* (L.) Batsch] throughout fruit development. The influence of deficit irrigation treatments on stem (*Ψ*_stem_) and leaf water potential, leaf relative water content, leaf stomatal conductance, and fruit growth was studied across the stages of double-sigmoidal fruit development in ‘September Bright’ nectarines. Fruit relative growth rate (RGR) and leaf relative pressure change rate (RPCR) were derived from FD and *p*_p_ to represent rates of water in- and outflows in the organs, respectively. Continuous RGR and RPCR dynamics were independently and jointly related to plant water status and environmental variables. The independent use of RGR and RPCR yielded significant associations with midday *Ψ*_stem_, the most representative index of tree water status in anisohydric species. However, a combination of nocturnal fruit and leaf parameters unveiled an even more significant relationship with *Ψ*_stem_, suggesting a changing behavior of fruit and leaf water flows in response to pronounced water deficit. In conclusion, we highlight the suitability of a dual-organ sensing approach for improved prediction of tree water status.

## Introduction

Precision irrigation is becoming a crucial management approach for environmentally and economically sustainable fruit tree production. The vast majority of fruit crops need irrigation supply as rainfall does not match crop water requirements (Stöckle et al., 2011; Snyder, 2017). In most cases of fruit crops cultivated in dry areas, rainfed agriculture is not sustainable and deficit irrigation (DI) is a reasonable strategy to improve water use efficiency. Fereres and Soriano (2007) highlighted the benefits of regulated DI as a strategy to reduce agricultural water use. The main purpose of regulated DI is to reduce irrigation at specific developmental stages of the crop with no or limited effects on yield. The use of DI in different phenological stages of fruit crops started in the 1980s by Chalmers et al. (1981, 1986). Today, water supply for DI treatments is often calculated as a fraction of crop evapotranspiration (ET_c_) (Naor, 2006; Paço et al., 2006) or weather-based modeling crop water requirements. Additional approaches rely on soil- or plant-based sensing. Irrigation management in nectarine was recently studied with regard to soil water content (Vera et al., 2019).

Plant physiological indicators of water deficit are predominantly subjected to changes in tissue water content and status rather than to soil water dynamics (Jones, 2004; Steppe et al., 2008). Moreover, to adequately represent soil spatial variability and wetted and non-wetted zones in irrigated crops, soil-based sensing requires the use of many sensors, making this approach costly and difficult. Therefore, a continuous assessment of plant water status indicators might provide the most precise information for irrigation management and automation. The advantage of plant-based methods over soil-based techniques resides in the fact that plants are an interface between soil and atmosphere (Fernández, 2017), being in the middle of the soil-plant-atmosphere continuum (SPAC). Therefore, precise automated irrigation management, in terms of the quantity and timing of water effectively required by plants, is likely to be highly associated to direct or indirect measurements of plant physiological indicators.

Midday stem water potential (*Ψ*_stem_) is one of the most widely used indicators of plant water status for irrigation scheduling in anisohydric plants (McCutchan and Shackel, 1992; Shackel et al., 1997; Naor, 2000). Conversely, Blanco-Cipollone et al. (2017) suggested the adoption of pre-dawn leaf water potential (*Ψ*_leaf_) as a suitable parameter for irrigation scheduling in isohydric species such as grapevine. Leaf relative water content (RWC) can also be used as a water deficit indicator (Lo Bianco and Scalisi, 2017; Mossad et al., 2018), although differently from water potential it does not give an indication of water energy status (Jones, 2007). Indicators of leaf water status may not be very useful in the early detection of plant water deficit in isohydric species (Jones, 2004), as their preventive stomatal closure preserves leaf turgor and leaf RWC.

A completely automated model for irrigation management in fruit crops is difficult to achieve, as responses to water deficit not only depend on environmental variables and soil water availability, but also on fruit tree phenology. In stone fruits (e.g., peach, nectarines, plums), tree water status and sink-source relationships differ in the three stages of the typical double sigmoidal fruit growth model (Connors, 1919; Chalmers and van den Ende, 1975), as shown in peach by DeJong and Goudriaan (1989). Consequently, DI applied at each of the stages of peach fruit growth affects vegetative and fruit growth differently, causing changes in final fruit size and composition (Li et al., 1989a). Fruit water exchanges follow skin transpiration, phloem and xylem streams, with different mechanisms linked to fruit growth stages (i.e., cell division, pit hardening, and cell enlargement), such as increasing transpiration and xylem inflow toward harvest (Marsal and Girona, 1997; Morandi et al., 2007a, 2010a). In peach, drought in early stages induces a relatively lower reduction of fruit development, compared to final stages, when cell enlargement occurs (Li et al., 1989a; Génard and Huguet, 1996).

A field direct, error-free, and continuous estimation of *Ψ*_stem_, *Ψ*_leaf_, or leaf RWC is not feasible yet in fruit tree crops, although stem psychrometers are currently being revived (Tran et al., 2015). The use of further plant-based technologies might represent a viable solution for the estimation of tree water status indicators. Trunk-based sensing such as sap-flow methods and dendrometry have been used for irrigation scheduling in peach and several other fruit crops (Fernández, 2017). Li et al. (1989b), Simonneau et al. (1993), and Goldhamer et al. (1999) successfully associated peach tree water status to stem diameter fluctuations obtained by dendrometers built on linear variable displacement transducers (LVDTs). In addition, Conejero et al. (2007) studied peach maximum trunk daily shrinkage and sap-flow signals for irrigation scheduling, suggesting that the former represents a more sensitive indicator of plant water deficit. Nevertheless, the use of stem/trunk diameter variations and sap flow for irrigation scheduling is questionable. Trunk diameter fluctuations are affected by plant age and size, crop load, environmental variables, and growth patterns (Fernández, 2017), whereas sap flow rates reflect transpiration dynamics, which are not only dependent on stomatal closure and aperture, but also on environmental variables (Jones, 2004).

The use of fruit- and leaf-based sensors to study tree water relations has also been reviewed in the literature (Jones, 2004; Fernández, 2017; Scalisi et al., 2017). Combined information obtained from fruit and leaf water continuous sensing may represent an innovative approach to determine sensitive indicators to water deficit. Changes in peach fruit water content in response to drought may be assessed with a model developed by Génard and Huguet (1996). The most common type of fruit-based sensor used to determine when trees enter water deficit conditions is based on LVDT technologies. Lang (1990) used LVDT sensors on apple fruit to emphasize the role of phloem, xylem, and transpiration on diameter changes over time. Similar sensors were used by Morandi et al. to study vascular flows in peach (Morandi et al., 2007a, 2010a), kiwifruit (Morandi et al., 2010b), and pear (Morandi et al., 2014). Fruit growth dynamics are a good indirect indicator of fruit water status (Fernandes et al., 2018), as dry matter accumulation is negligible on a daily scale (Blanke and Lenz, 1989). Fruit growth dynamics however can be influenced by growth stage and crop load. In peach, fruit water dynamics vary across the season, with maximum transpiration at fruit cell enlargement (Morandi et al., 2010a). Consequently, the use of fruit gauges alone may not be a reliable indicator of water deficit in trees.

Leaf-based sensing technologies mainly adopt leaf thickness sensors and pressure probes. The continuous outputs of the former were related to leaf RWC (Burquez, 1987), although their long-term use is not feasible as they commonly injure leaves after short time (Zimmermann et al., 2008). Therefore, recently, a less invasive leaf pressure probe for the continuous determination of leaf water status (Zimmermann et al., 2008) has entered the market. These so-called leaf patch clamp pressure (LPCP) probes can be used to assess water stress for irrigation scheduling, as they respond to leaf turgor pressure, which has an important role in *Ψ*_leaf_. Most of the initial studies with LPCP probes were carried out on olive (Fernández et al., 2011; Ehrenberger et al., 2012; Rodriguez-Dominguez et al., 2012; Padilla-Díaz et al., 2016) because the thick leaves of this species better suit the prolonged use of sensors. Olive is cultivated in dry or semi-dry regions with limited or no irrigation water supply. LPCP probes were also related to plant water status indicators in other fruit crops, such as banana (Zimmermann et al., 2010), grapevine (Rüger et al., 2010), clementine (Ballester et al., 2017), and persimmon (Ballester et al., 2017; Martínez-Gimeno et al., 2017). However, as for fruit sensors, the use of LPCP probes alone can only give partial information on whole plant water status, unless many sensors are used on a tree. This is particularly due to different leaf initial conditions depending on age (especially in evergreen species) and exposure to light within the canopy. Even accepting the quality of the data, a further need to test LPCP probes on species with thinner leaves (e.g., stone fruits) arises, as their prolonged use might damage leaf cuticle and alter measurements (Scalisi et al., 2017). As mentioned above, the use of a single type of sensors can only provide partial information on tree water status. Most of C3 fruit trees exchange water with the surrounding atmosphere by means of transpiring fruit and leaves.

This study aimed to investigate the relationship of *Ψ*_stem_ and other plant water status indicators to continuous fruit size and leaf turgor pressure dynamics in nectarine trees [*Prunus persica* (L.) Batsch] subjected to DI at each of the individual stages of fruit growth. The main hypothesis was that the combined information from fruit and leaves (i.e., the transpiring organs) provides more powerful information than individual indicators to determine plant water status on a continuous basis for adoption of precision irrigation management.

## Materials and Methods

### Experimental Design

The experiment was carried out in summer 2017/18 on late ripening ‘September Bright’ nectarine trees grafted on “Elberta” rootstock at the research station of Agriculture Victoria, Tatura, Australia (36°26′7.2″ S and 145°16′8.4″ E, 113 m a.s.l.). Within the experimental site, 144 4-year-old trees trained to an open Tatura system with 4.5 m × 1 m spacing (i.e., 2,222 trees/ha) were selected. Trees were disposed along N-to-S oriented rows. The soil was a clay-loam and trees were regularly fertigated according to conventional protocols. Fruit thinning and summer pruning were carried out at 43 and 125 days after full bloom (DAFB), respectively.

The typical double-sigmoidal fruit growth pattern was characterized by measurements of fruit diameter in control trees at weekly intervals from shuck fall to harvest. Growth stages were divided as follows: a cell division stage (I), a pit hardening stage (II), and a cell expansion stage (III) further subdivided into two equal phases of about a month each (previous year observations), with the first (IIIa) starting when fruit cells re-establish a strong sink power after stage II, and the second being the final period of sugar accumulation and chlorophyll degradation (stage IIIb). Four different DI levels, namely 100% of crop evapotranspiration (ET_c_, control), 40% of ET_c_ (DI-40), 20% of ET_c_ (DI-20), and 0% of ET_c_ (DI-0) were applied from the beginning to the end of each fruit growth stage, using a drip irrigation system. At stage I, an initial fertigation resulted in additional 13 mm of water added to the DI-0 treatment. The experimental design included six replications in a randomized complete block design, each with two tree orientations (East and West) per treatment and fruit growth stage; measurement trees were separated by buffer trees and rows. At stage IIIb, the DI-40 treatment was not considered, due to limited number of trees available. Canopy orientation was also considered in the design, including West- and East-oriented trees of the open Tatura system. This was particularly helpful to explain different responses among trees due to light interception at different times of the day.

Meteorological data were collected using a weather station located in the experimental field. Solar radiation was measured using a silicon pyranometer (SK01D, Carter-Scott Design, Brunswick, Australia). Relative humidity (RH) and temperature (T) measurement were based on the combination of a capacitive thin film polymer sensor HUMICAP®180 and resistive platinum sensors (HMP 45A-T, Vaisala, Finland). Rainfall was measured using a TB3A rain gauge (Hydrological Services PL, Warwick Farm, Australia). Wind speed was measured with a wind transmitter (Model No. 4.3519.00.000, Thies Clima, Göttingen, Germany). Measurements were stored at 10-min intervals in a 6004C-21 STARLOG data logger (Unidata, O’Connor, Australia).

Reference evapotranspiration (ET_0_) and vapor pressure deficit (VPD) were calculated using the methods described by Allen et al. (1998). Cumulative average daily VPD (∑_VPD_) and cumulative ET_0_ (∑_ET0_) were obtained by the summation of average daily VPD and total daily ET_0_, respectively, for each fruit growth stage. Average daily vapor pressure deficit (*μ*_VPD_) and average daily reference evapotranspiration (*μ*_ET0_) were obtained by dividing respectively ∑_VPD_ and ∑_ET0_, by the number of days in each fruit development stage. Crop evapotranspiration (ET_c_) was estimated based on Equation 1.

(1) $${ET}_{c}=\left(K_{cb}\times {ET}_{0}\right)+\left(K_{e}\times {ET}_{0}\right)$$

where *K*_cb_ is the crop basal coefficient, calculated as 1.05 × EAS (effective area of shade) (Goodwin et al., 2006), and *K*_e_ is a soil evaporation coefficient of 0.1 in accordance with Bonachela et al. (2001).

### Fruit Size and Tree Water Relations

#### Fruit Size

Fruit diameters were measured at weekly intervals in the morning of stages I, II, IIIa, and IIIb, using a Calibit digital caliper (HK Horticultural Knowledge srl, Bologna, Italy). Measurements were carried out on three fruits per tree for each irrigation treatment and canopy orientation, for a total of 36 fruits on 12 trees (two in each of the six blocks) for each irrigation treatment. Data from differently oriented trees, i.e., East and West, were pooled together as fruit diameters were not significantly different at any of the stages considered.

#### Water Potential

A pressure chamber (3000 Scholander Plant Water Status Consol, ICT International, Armidale, Australia) was used for the measurements of *Ψ*_stem_ and *Ψ*_leaf_ according to Turner (1988). Mature, fully expanded leaves were covered with foil-laminate bags 2 h prior to each *Ψ*_stem_ measurement, except for pre-dawn. Midday *Ψ*_stem_ was determined at weekly intervals in all the stages of fruit growth on three leaves of the two trees (East- and West-oriented) per treatment in one of the six blocks. The block was randomly selected at the beginning of the experiment and used throughout the experimental period for water potential and other water status indicators. Daily curves from pre-dawn to 19:00 h were plotted using *Ψ*_stem_ and *Ψ*_leaf_ data collected at three-hour intervals. Measurements for daily curve characterization were carried out on a single day for each growth stage (I at 50 DAFB, II at 92 DAFB, IIIa at 132 DAFB and IIIb at 155 DAFB) for *Ψ*_stem_ and only in stage IIIa (132 DAFB) and IIIb (155 DAFB) for *Ψ*_leaf_.

#### Leaf Relative Water Content

Leaf RWC was obtained using the method described by Barrs and Weatherley (1962). Mature, fully expanded leaves were collected, sealed in plastic bags, and transported to the laboratory for fresh weight (FW) determination. Turgid weight (TW) was obtained after immersing leaves in deionized water for 24 h at 4°C. Subsequently, leaves were dried in an oven at 60°C until constant weight (2–3 days) to estimate dry weight (DW). Leaf RWC was calculated as shown in Equation 2.

(2) $$RWC=\left(FW-DW\right)/\left(TW-DW\right)\;\times \;100$$

Leaf RWC was determined at 3-hour intervals on the same days and trees as *Ψ*_stem_ and *Ψ*_leaf_.

#### Leaf Stomatal Conductance

A Delta-T AP4 dynamic porometer (Delta-T Devices LTD, Cambridge, UK) was used to determine leaf stomatal conductance (*g*_s_). Mid-morning (10–11 am) measurements of *g*_s_ were undertaken at weekly intervals in all the stages of fruit growth on four leaves of the two trees (East- and West-oriented) for each treatment, and in the same block selected for water potential measurements. Stage-related *g*_s_ daily curves were obtained from three leaves on the same days and trees as *Ψ*_stem_ and *Ψ*_leaf_ determination.

### Fruit Diameter and Leaf Turgor Pressure Continuous Sensing

Fruit diameter (FD) was determined continuously with the LVDT-based fruit gauges described by Morandi et al. (2007b) connected to CR-1000 data loggers (Campbell scientific, Inc., Logan, US). Concurrently, leaf-mounted LPCP probes (Yara International, Oslo, NO) were used to track leaf turgor pressure dynamics using the attenuated pressure of leaf patches (*p*_p_), an index which is inversely related to leaf cell turgor pressure (*p*_c_), as described by Zimmermann et al. (2008). Data from both sensors were recorded at 15-min intervals for a week at each of the growth stages (starting at 48, 86, 127, and 155 DAFB for stage I, II, IIIa, and IIIb, respectively) in one of the blocks within the experimental orchard. The block selected was the same used for *g*_s_, *Ψ*_stem_, *Ψ*_leaf_, and leaf RWC measurements. Two fruit gauges and LPCP probes were mounted on each West- and East-oriented tree under all irrigation treatments, at mid-canopy height and in nearby positions. Before the actual week of measurements, a preliminary 3-day comparison test between East- and West-oriented trees was carried out to verify if canopy orientation had an effect on sensors’ outputs. Data from East- and West-oriented trees were compared using daily relative standard deviations (RSD), mean, sum, max, and min.

Raw data obtained from fruit gauges and LPCP probes were smoothed using a 15-point convoluted spline function (Savitzky and Golay, 1964). Subsequently, FD and *p*_p_ values were standardized by using *z*-scores [i.e., *z* = (*x* − mean)/standard deviation] to enable the comparison among fruits or leaves, respectively, which had different characteristics when the sensors were attached (i.e., fruit diameter and leaf turgor pressure). Resulting *z*-scores show positive and negative values as they are calculated assuming a distribution with a mean of 0 and a standard deviation of 1, in the specific time interval used for the calculation. Once FD and *p*_p_ were standardized, it was possible to average more sensors’ output on the same tree and compare different treatments. Furthermore, the second derivatives of fruit diameter and *p*_p_ were calculated to determine fruit relative growth rate (RGR) and leaf relative pressure change rate (RPCR), as shown in Equations 3 and 4, respectively. Second derivatives were not standardized as they are calculated based on the previous FD and *p*_p_, allowing possible comparisons among outputs from different sensors.

(3) $$RGR=\left[\ln\;\left({FD}_{2}\right)-\ln\;\left({FD}_{1}\right)\right]/t_{2}-t_{1}$$

(4) $$\mathrm{RPCR}=\left[\ln\;\left(p_{p2}\right)-\ln\;\left(p_{p1}\right)\right]/t_{2}-t_{1}$$

where FD_2_ and FD_1_ correspond to FD at time 2 (*t*_2_) and 1 (*t*_1_), and *p*_p2_ and *p*_p1_ correspond to *p*_p_ at time 2 (*t*_2_) and 1 (*t*_1_), respectively. The time interval between *t*_2_ and *t*_1_ was 15 min.

Diel, diurnal, and nocturnal variance of sensors’ outputs was expressed as relative standard deviation (RSD = standard deviation/ǀmeanǀ), to allow comparison among variances of different units (i.e., FD/*p*_p_ and RGR/RPCR). In addition, also diel, diurnal, and nocturnal statistical parameters from data series were calculated for the variables considered (i.e., maximum, minimum, and sum values) in order to find the best predictor of midday *Ψ*_stem_.

A small portion of data (<5%) from sensors that either caused damage to leaves or fruit or that were displaced by strong wind was not considered in the analysis.

### Statistical Analysis

Statistical analysis was carried out using SYSTAT procedures (Systat software Inc., Chicago, USA). Analysis of variance was performed based on the randomized block design, using irrigation treatments, canopy orientation, and time as factors, and, when appropriate, means were compared by Tukey’s multiple range test and honestly significant difference (HSD). Canopy orientation often did not influence results or interact with other factors. Main interactions were found when using irrigation treatments and time as factors. Sigmaplot procedures (Systat software Inc., Chicago, USA) were used for linear and multiple linear regression analyses in order to associate continuous sensors’ output to plant water status indicators.

## Results and Discussion

### Fruit Developmental Stages, Weather Conditions, and Crop Water Supply

The typical double sigmoidal fruit development pattern was observed in control fruit, and stages I, II, IIIa, and IIIb lasted 36, 50, 29, and 31 days, respectively (Table 1).

**Table 1:** Total rainfall, average daily vapor pressure deficit (*μ*_VPD_), cumulative average daily vapor pressure deficit (∑_VPD_), average daily reference evapotranspiration (*μ*_ET0_), cumulative reference evapotranspiration (∑_ET0_), and irrigation volumes for trees irrigated to 100% (control), 40% (DI-40), 20% (DI-20), and 0% (DI-0) of crop evapotranspiration at each of the fruit growth stages.

Fruit growth stage	Duration (days)	Rainfall (mm)	*μ*_VPD_ (kPa)	∑_VPD_ (kPa)	*μ*_ET0_ (mm)	∑_ET0_ (mm)	Irrigation volume (mm)			
							Control	DI-40	DI-20	DI-0
I	36	27	0.75	27.2	4.92	177	63	28	16	13^y^
II	50	141	1.14	50.1	6.18	309	78	27	15	0
IIIa	29	35	1.48	42.8	7.28	211	75	32	16	0
IIIb	31	3	1.26	40.4	6.13	190	83	n.a.^z^	19	0
Total	146	243	1.16	160.5	6.15	848	315	91	54	13

y *Fertigation*.

z *Not applicable*.

**Figure 1:**
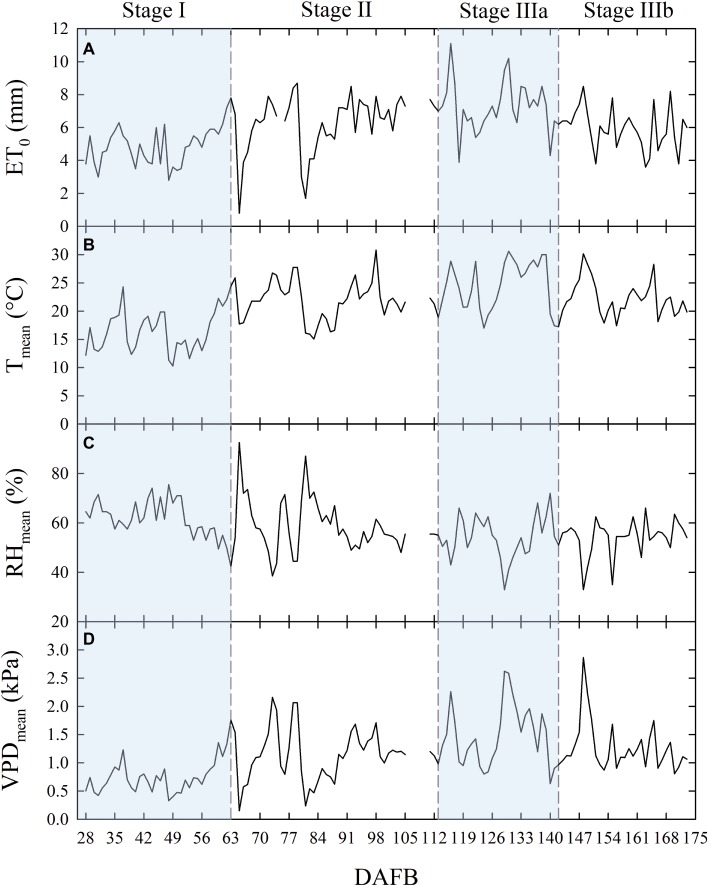
Daily total reference evapotranspiration [ET_0_ **(A)**], mean temperature [*T*_mean_ **(B)**], mean relative humidity [RH_mean_ **(C)**], and mean vapor pressure deficit [VPD_mean_ **(D)**] along the considered four stages of fruit growth in days after full bloom (DAFB). Missing data from 106 to 110 DAFB.

Temperature (T), relative humidity (RH), ET_0_, and VPD recorded from 27 to 173 days DAFB are shown in Figure 1. The gap in the data from 106 to 110 DAFB was due to a battery discharge. In stage II, frequent and abundant precipitations (Table 1) led to relatively low T (Figure 1B) and high RH (Figure 1C) (i.e., from 78 to 89 DAFB). Maximum ET_0_ occurred at stage IIIa (Figure 1A, Table 1), driven by a combination of high T and low RH which caused a rise in VPD (Figure 1D). Precipitations progressively decreased toward the end of stage IIIb (Table 1). Trees received more water in stage II, due to more rainfall and a longer duration compared to other fruit growth stages (Table 1). Overall, the crop water supply (irrigation + rainfall) during fruit development stages for control trees was equal to 558 mm (Table 1). The highest ∑_VPD_ and ∑_ET0_ occurred in stage II (Table 1), due to the relatively higher duration of this stage compared to others and to the abundant crop water supply. Indeed, when the latter were weighed on the number of days (*μ*_VPD_ and *μ*_ET0_) the highest values were found in stage IIIa (Table 1).

### Fruit Size and Tree Water Relations

#### Fruit Size

**Figure 2:**
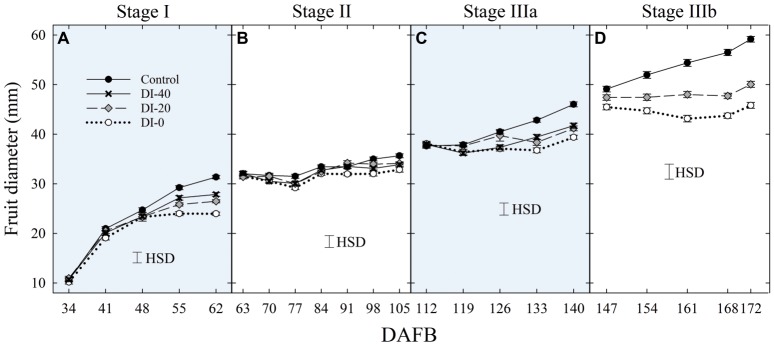
Fruit diameter at stage I **(A)**, II **(B)**, IIIa **(C)**, and IIIb **(D)** of ‘September Bright’ nectarine fruit growth. Measurements done with a fruit caliper on trees irrigated to 100% (control), 40% (DI-40), 20% (DI-20), and 0% (DI-0) of crop evapotranspiration. Timeline expressed in days after full bloom (DAFB). Error bars represent standard errors of means (*n* = 36). Significant differences determined with analysis of variance and Tukey’s honestly significant difference (HSD, *p* < 0.05).

No significant difference in fruit size determined with digital caliper measurements was found between East- and West-oriented trees (data not shown), thus data from the two sides were pooled together. At stage I, fruit diameter was significantly reduced by DI at 55 DAFB, with DI-20 and DI-40 inducing similar reductions and intermediate between the control and DI-0 (Figure 2A). At stage II, during pit hardening, fruit diameter was only slightly affected by DI treatments, and significant differences only emerged at the end of the stage between control and DI-0 trees (Figure 2B). At stage IIIa, DI induced fruit diameter reductions similar to those at stage I, with all DI treatments showing similar reductions compared to the control (Figure 2C). Finally, DI caused the highest reduction of fruit growth at stage IIIb (Figure 2D). Results from DI in stages I, II, IIIa, and IIIb are in line with findings in peach from Li et al. (1989a) and Génard and Huguet (1996), and in nectarines from Naor et al. (1999, 2001).

#### Water Potential

**Figure 3:**
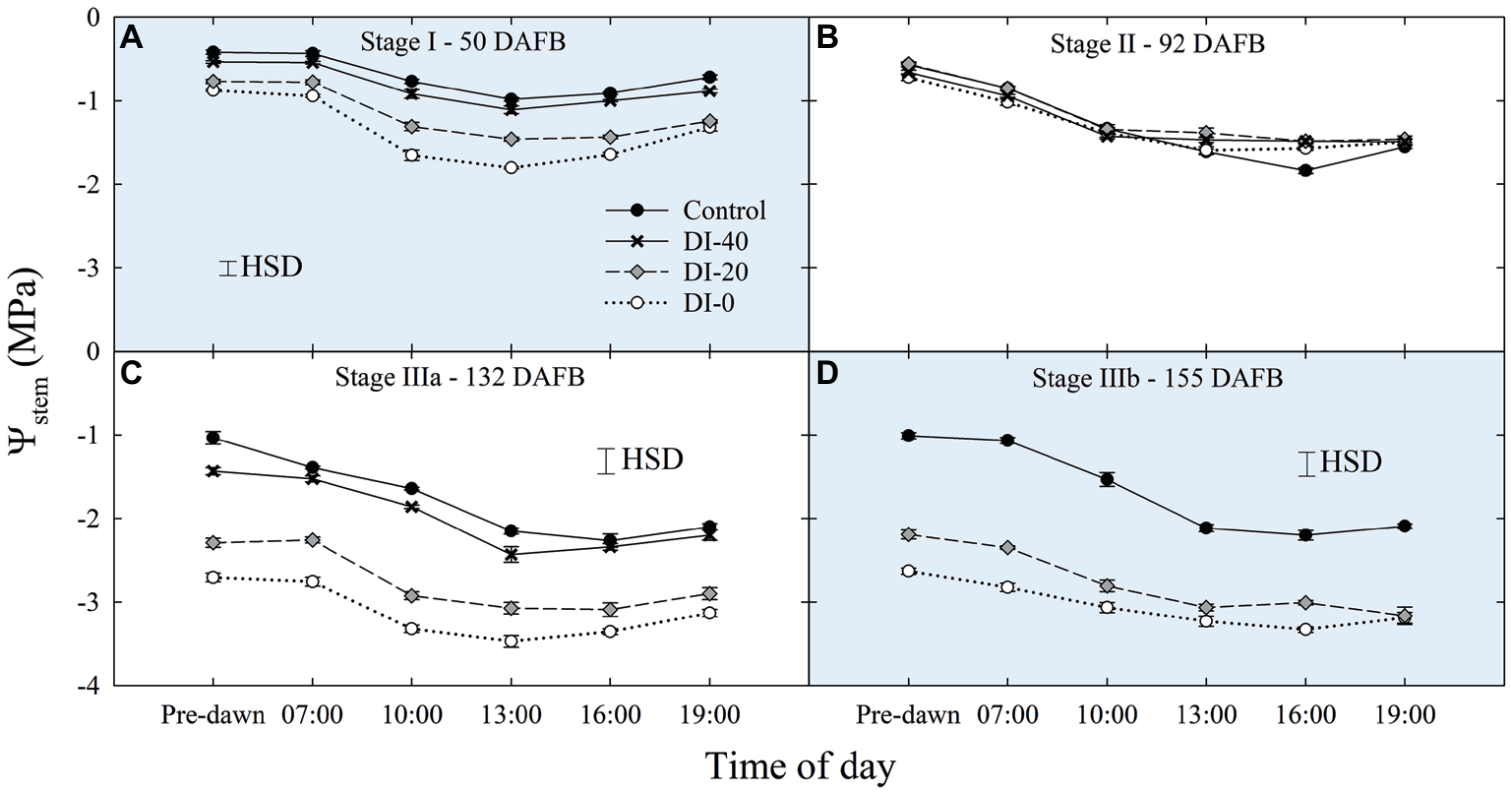
Daily curves of stem water potential (*Ψ*_stem_) at stages I **(A)**, II **(B)**, IIIa **(C)**, and IIIb **(D)** of ‘September Bright’ nectarine fruit growth. Trees irrigated to 100% (control), 40% (DI-40), 20% (DI-20), and 0% **(DI-0)** of crop evapotranspiration. Error bars represent standard errors of means (*n* = 6). Significant differences determined with analysis of variance and Tukey’s honestly significant difference (HSD, *p* < 0.05).

When water potentials from East- and West-oriented trees were compared, no statistically significant differences were found, thus data from the two sides were pooled together. Daily curves of *Ψ*_stem_ highlighted a relevant and gradual separation among irrigation treatments at solar noon measurements, except for stage II (Figure 3), a further evidence of the suitability of midday *Ψ*_stem_ as an indicator of plant water deficit, as previously shown by Naor et al. (1999). The lack of an effect of DI on *Ψ*_stem_ at stage II might be related to the abundant precipitations which occurred during this phase (Table 1).

**Figure 4:**
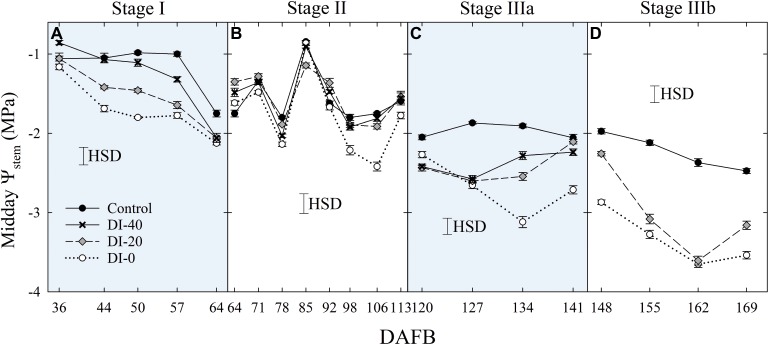
Midday stem water potential (*Ψ*_stem_) at stages I **(A)**, II **(B)**, IIIa **(C)**, and IIIb **(D)** of ‘September Bright’ nectarine fruit growth. Trees irrigated to 100% (control), 40% (DI-40), 20% (DI-20), and 0% (DI-0) of crop evapotranspiration. Timeline expressed in days after full bloom (DAFB). Error bars represent standard errors of means (*n* = 6). Significant differences determined with analysis of variance and Tukey’s honestly significant difference (HSD, *p* < 0.05).

Similarly, when weekly midday *Ψ*_stem_ was considered, the effect of DI treatments increased gradually with fruit growth, reaching the most marked reductions at the end of stage IIIb (Figure 4). Overall, *Ψ*_stem_ decreased in all treatments along the fruit development period, suggesting a likely higher tree water consumption in the latest stages of high assimilate demand (Chalmers and Wilson, 1978). Even in this case, minor or no effects were found at stage II, although in the second half, decreasing precipitations (data not shown) unveiled a drop of midday *Ψ*_stem_ in DI-0 trees (Figure 4B). A steeper decrease of midday *Ψ*_stem_ at stage II was also found by Fereres and Soriano (2007) in peach.

**Figure 5:**
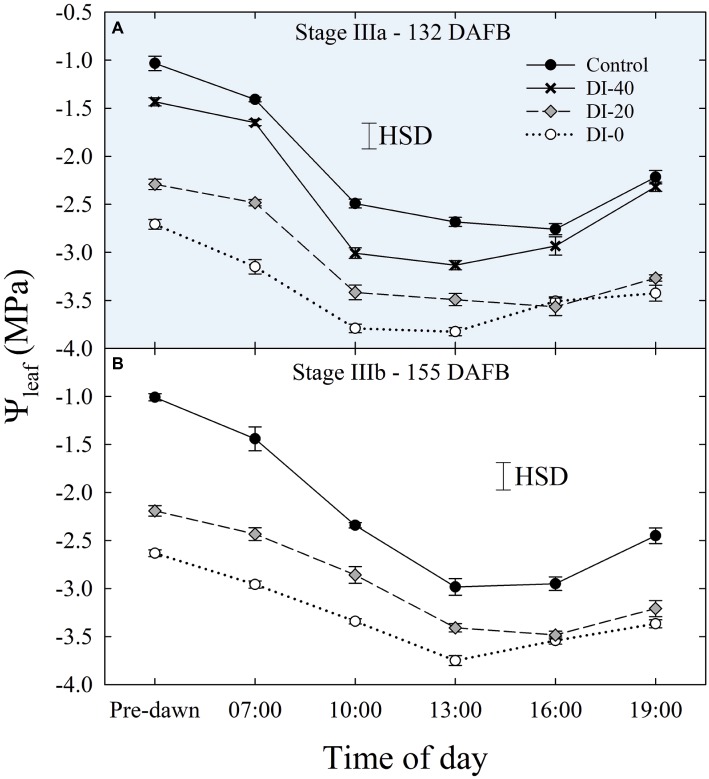
Daily curves of leaf water potential (*Ψ*_leaf_) at stages IIIa **(A)** and IIIb **(B)** of ‘September Bright’ nectarine fruit growth. Trees irrigated to 100% (control), 40% (DI-40), 20% (DI-20), and 0% (DI-0) of crop evapotranspiration. Error bars represent standard errors of means (*n* = 6). Significant differences determined with analysis of variance and Tukey’s honestly significant difference (HSD, *p* < 0.05).

Daily measurements of *Ψ*_leaf_ carried out only in stage IIIa and IIIb (Figures 5A,B), and concomitantly with *Ψ*_stem_, showed typical patterns with lowest values around solar noon. As expected, *Ψ*_leaf_ resulted in slightly lower values than *Ψ*_stem_, in accordance with the water potential gradient along the SPAC. DI-0 trees reached the lowest *Ψ*_leaf_ of −3.82 and −3.75 MPa in stages IIIa and IIIb, respectively (Figure 5).

#### Leaf Relative Water Content

**Figure 6:**
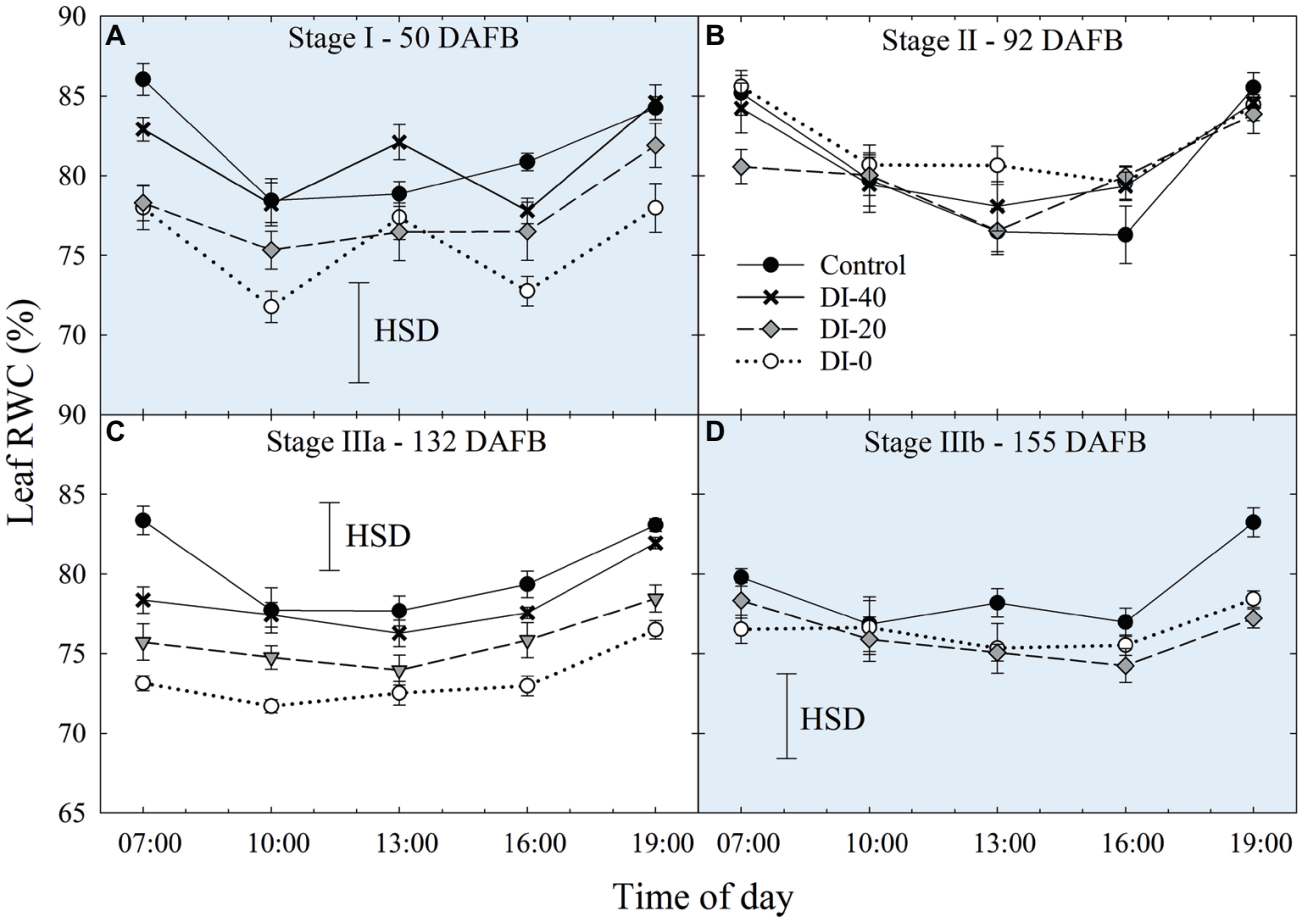
Daily curves of leaf relative water content (RWC) at stages I **(A)**, II **(B)**, IIIa **(C)**, and IIIb **(D)** of ‘September Bright’ nectarine fruit growth. Trees irrigated to 100% (control), 40% (DI-40), 20% (DI-20), and 0% (DI-0) of crop evapotranspiration. Error bars represent standard errors of means (*n* = 6). Significant differences determined with analysis of variance and Tukey’s honestly significant difference (HSD, *p* < 0.05).

Daily curves of leaf RWC obtained from measurements carried out at all the fruit development stages and on all the irrigation treatments did not highlight differences among West- and East-oriented trees (data not shown), thus data from the two sides were pooled together. At stage I, leaf RWC varied greatly, showing erratic effects of DI (Figure 6A). At stage II, irrigation treatment and time of day had no significant effect on leaf RWC (Figure 6B). However, leaf RWC was found gradually lower along the irrigation treatment gradient at stage IIIa (Figure 6C), where the maximum differences between the two extreme treatments, control and DI-0, occurred at mid-morning and mid-afternoon. Ultimately, at stage IIIb, differences among treatments were once again nonsignificant, except for the measurement at 19:00 h (Figure 6D). Therefore, leaf RWC cannot be considered as sensitive as *Ψ*_stem_ and *Ψ*_leaf_ for nectarine water status determination, mainly because the variability of RWC among leaves is high and results in nonsignificant effects of DI (i.e., HSD in Figure 6). This variability is determined by intrinsic leaf characteristics such as age and competition with other leaves on the same shoot. In addition, when trees cope with high water deficit, stomata tend to close (as suggested by our *g*_s_ results shown in the next paragraph) and leaf RWC is readjusted in accordance with osmotic gradients. This explains why, despite daily *Ψ*_stem_ and *Ψ*_leaf_ being similar in DI-0 trees in stages IIIa and IIIb (Figures 3C,D, 5A,B), average daily leaf RWC in the same trees was slightly higher in stage IIIb (76.5% ± 0.49) than in stage IIIa (73.4% ± 0.73), with the former not showing significant changes from 7:00 to 19:00 h (Figure 6D).

#### Leaf Stomatal Conductance

**Figure 7:**
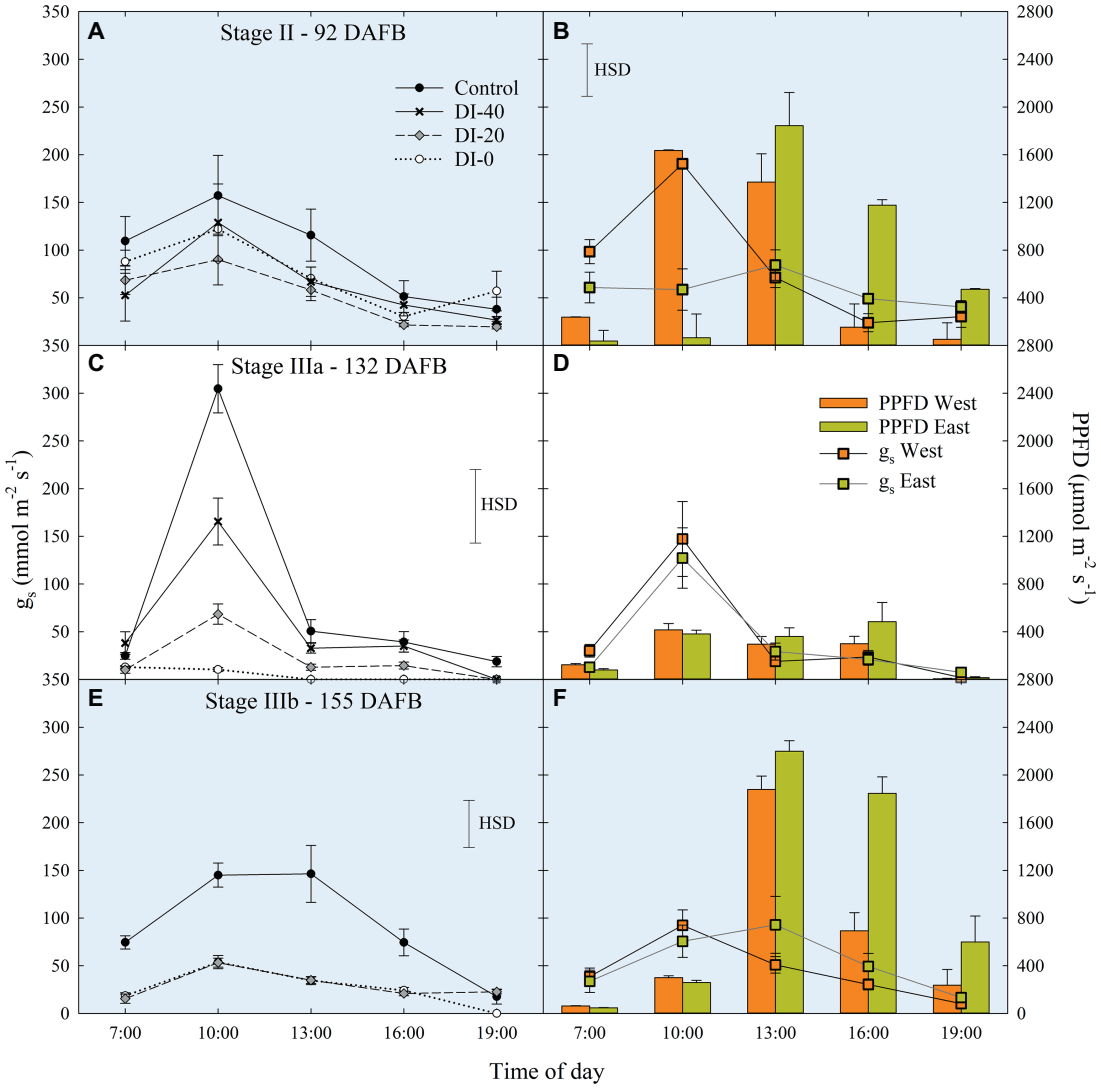
Daily curves of leaf stomatal conductance (*g*_s_) at stages II **(A)**, IIIa **(C)**, and IIIb **(E)** of ‘September Bright’ nectarine fruit growth, and in West- and East-oriented trees [Stage II **(B)**, IIIa **(D)**, IIIb **(F)**]. Trees irrigated to 100% (control), 40% (DI-40), 20% (DI-20), and 0% (DI-0) of crop evapotranspiration. Bars in panels **(B)**, **(D)**, and **(F)** show means of photosynthetic photon flux density (PPFD) for West- and East-oriented trees. Bars represent standard errors of means (irrigation treatment *n* = 6; canopy orientation *n* = 12). Significant differences determined with analysis of variance and Tukey’s honestly significant difference (HSD, *p* < 0.05). The HSD bars in panels **(B,D,F)** represent only differences in *g*_s_, and not in PPFD (only used as a reference).

Data of *g*_s_ are available since stage II of fruit development due to instrument malfunctioning. At stage II, no significant differences in daily *g*_s_ were found among irrigation treatments (Figure 7A). When maximum stomatal aperture occurred (mid-morning), there was a significant influence of canopy orientation, resulting in higher *g*_s_ in leaves of West-oriented trees (Figure 7B), as they intercepted greater PAR than East trees. After noon, an overall partial closure of stomata induced a consequential reduction of *g*_s_ in all the treatments. At stage IIIa, control irrigated trees expressed a *g*_s_ higher than 300 mmol m^−2^ s^−1^ in the morning, whereas DI-0 trees barely opened their stomata (about 10 mmol m^−2^ s^−1^) in response to high water deficit conditions (Figure 7C). Differently from stage II, no differences were found between West- and East-oriented trees, because measurements were done on a cloudy day (Figure 7D). At stage IIIb, leaves of control trees had higher *g*_s_ compared to DI-20 and DI-0 trees, which instead showed similar *g*_s_ levels (Figure 7E). In addition, even in the case of stage IIIb daily curve, a cloudy morning concealed the effect of canopy orientation, and the increase of photosynthetic photon flux density (PPFD) caused by the disappearance of clouds after solar noon was not sufficient to show differences between West- and East-oriented trees (Figure 7F).

**Figure 8:**
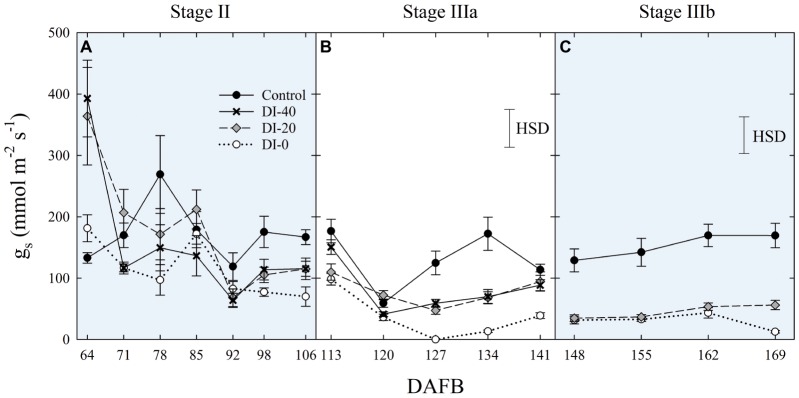
Mid-morning leaf stomatal conductance (*g*_s_) at stages II **(A)**, IIIa **(B)**, and IIIb **(C)** of ‘September Bright’ nectarine fruit growth. Trees irrigated to 100% (control), 40% (DI-40), 20% (DI-20), and 0% (DI-0) of crop evapotranspiration. Timeline expressed in days after full bloom (DAFB). Error bars represent standard errors of means (*n* = 6). Significant differences determined with analysis of variance and Tukey’s honestly significant difference (HSD, *p* < 0.05).

When measured at weekly intervals, *g*_s_ showed no differences among irrigation treatments at stage II (Figure 8A), whereas DI treatments reduced mid-morning stomatal aperture in the second half of stage IIIa (Figure 8B). Only at stage IIIb, leaves from control trees consistently kept their *g*_s_ higher than leaves from DI-20 and DI-0 trees (Figure 8C). At this stage, after reaching a severe water deficit, DI-0 and DI-20 trees limited their gas exchanges to minimal levels, and likely DI induced a reduction of phloem flows toward fruit, or increasing xylem backflow, following water potential gradients along the vascular path. To confirm this hypothesis, studies on isolated xylem and phloem contribution to ‘September Bright’ fruit growth might be carried out on girdled and detached fruit, as explained by Lang (1990). Therefore, a reduction of leaf gas exchanges might partially explain the poor, nonsignificant increase of fruit size observed in DI-0 (Figure 2D). Overall, *g*_s_ data at mid-morning were found to be a representative indicator of plant water deficit, as that is the time of highest leaf transpiration and maximum evidence of partial stomatal closure in response to water deficit.

#### The Interdependency of Plant Water Status Indicators

Among the others, *Ψ*_stem_ can be considered as the most sensitive indicator of plant water status in nectarines, and it is strictly related to other water status indices along the SPAC (e.g., *Ψ*_leaf_ and external VPD) and to the regulation of stomatal opening, expressed in terms of *g*_s_. Leaf RWC has also been linked to *Ψ*_stem_ as shown by Koide et al. (1989), although results of this study were not always in line. Indeed, leaf RWC was not found to be a sensitive measurement to highlight differences among irrigation treatments, especially at stages I, II, and IIIb of fruit development (Figures 6A,B,D). In our case, the strongest association between leaf RWC and *Ψ*_stem_ occurred at pre-dawn, when water potential and water content were in equilibrium (data not shown).

The combined interdependency of VPD, *Ψ*_leaf_, *g*_s_, and leaf RWC with *Ψ*_stem_ was tested analyzing data extrapolated from daily curves from all the fruit growth stages. Data were pooled together and associated to *Ψ*_stem_ through a multiple linear regression model. Stomatal aperture and closure dynamics are known to be regulated by leaf RWC and *Ψ*_leaf_ among other factors, which in turn are influenced by VPD and strictly related to *Ψ*_stem_. Leaf RWC is adjusted responding to *Ψ*_stem_ and VPD gradients. More water can flow toward leaves in order to maintain higher *Ψ*_leaf_, stomatal aperture, and photosynthetic activity. Therefore, we expected to find the strongest association of *Ψ*_stem_ with *Ψ*_leaf_, followed by decreasingly tight associations with *g*_s_, VPD, and leaf RWC, respectively. However, leaf RWC resulted to be nonsignificant in a first backward stepwise regression model (*p* = 0.98), and it was excluded from the final outcome. Minor leaf RWC changes on a daily scale (Figure 6) may explain the absence of a relationship with *Ψ*_stem_. In the obtained multiple linear regression model, *Ψ*_stem_ was predicted from a linear combination of *Ψ*_leaf_, *g*_s_, and VPD (*R*^2^ = 0.867, *p* < 0.001, S.E. = 0.240), as shown in Equation 5.

(5) $$\begin{array}{l} \Psi_{\mathrm{stem}}=-0.311+\left(0.882\;\times \;\Psi_{\mathrm{leaf}}\right)+\left(0.004\;\times \;g_{s}\right)\\ \;+\left(0.077\times VPD\right) \end{array}$$

Our results are in line with findings in nectarines and other woody species (Naor, 1998), where *Ψ*_stem_ was found to be related to leaf stomatal conductance (*g*_s_) and *Ψ*_leaf_.

### Fruit Diameter and Leaf Turgor Pressure Continuous Sensing

**Figure 9:**
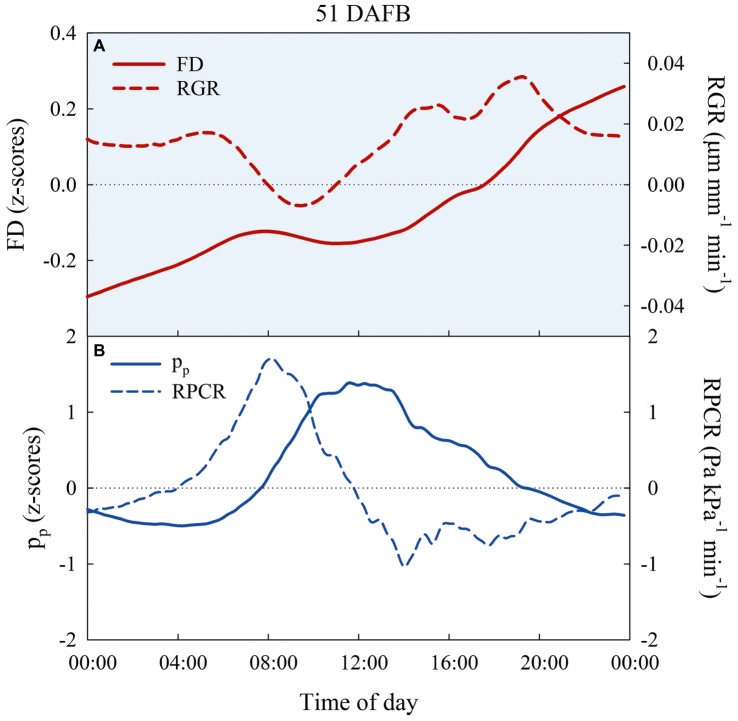
Diel trends of fruit diameter (FD, *n* = 3) and fruit relative growth rate (RGR, *n* = 3) **(A)**, attenuated pressure of leaf patches (*p*_p_) and leaf relative pressure change rate (RPCR) **(B)** in control irrigated trees at 51 days after full bloom (DAFB), at stage I of ‘September Bright’ nectarine fruit growth.

The preliminary trial on FD, *p*_p_, RGR, and RPCR responses of East- and West-oriented trees did not show any significant effect of canopy orientation. Consequently, for each fruit growth stage, FD and *p*_p_ data, as well as their derivatives (i.e., RGR and RPCR), from East- and West-oriented trees were pooled together for each irrigation treatment. *z*-scores ranged from negative to positive in the 24-h intervals selected (Figures 9A,B). In control trees, FD showed an expected nocturnal increase with a diurnal lag phase during stages I, IIIa, and IIIb. Figure 9A shows FD and RGR in a representative day at stage I (51 DAFB). In the warmest hours of the day, *p*_p_ increased, being the inverse of *p*_c_, as leaf turgor pressure was lost, and a peak of RPCR was observed in the first part of the morning (Figure 9B).

Initially, FD and *p*_p_ values, corresponding to the time of spot measurements of *Ψ*_stem_, *Ψ*_leaf_, *g*_s_, and leaf RWC from daily curves, were considered to determine whether any significant linear relationships occurred. Pearson’s correlation analyses emphasized in most cases, no significant linear correlation at all between FD and the water status indices, except for the association between FD and *Ψ*_leaf_ with a low correlation coefficient (Table 2). The inverse relationships with the highest correlation coefficients were found between *p*_p_ and leaf water status indices. The highest correlation coefficient was found between *p*_p_ and *Ψ*_leaf_, due to the high influence of leaf turgor pressure on the total *Ψ*_leaf_. The use of FD and *p*_p_ *per se* to find significant relationships with plant water status indices is likely to hide information as there is an intrinsic delay in the adjustment of water in tissue in response to plant water deficit. Hence, the rates at which FD and *p*_p_ change over time are likely to be more strictly related to water potential gradients in particular. Therefore, RGR and RPCR can be used to smooth delay of fruit and leaf responses to water deficit over time. Besides, the use of continuous data from leaves or fruit alone might not provide appropriate information on plant water status. When considered in isolation, data from fruit diameter changes are influenced by fruit development stage and fruit growth, while data of leaf turgor pressure may not be directly related to water balance in the other main organs capable of transpiration. As a consequence, the association of RGR and RPCR dynamics can highlight a ratio of fruit and leaf water exchanges which might reflect more precisely plant water status.

**Table 2:** Pearson’s correlation coefficients for fruit diameter (FD) and attenuated leaf patch clamp pressure (*p*_p_) vs. plant water status (PWS) indicators: stem water potential (*Ψ*_stem_), leaf water potential (*Ψ*_leaf_), leaf stomatal conductance (*g*_s_), and leaf relative water content (RWC) for all fruit growth stages.

PWS indicator	FD (*z*-scores)	*p*	*n*	*p*_p_ (*z*-scores)	*p*	*n*
*Ψ*_stem_ (MPa)	−0.103	0.184	168	−0.320	<0.001	180
*Ψ*_leaf_ (MPa)	−0.296	0.009	78	−0.645	<0.001	84
RWC (%)	−0.156	0.066	140	−0.442	<0.001	150
*g*_s_ (mmol m^−2^ s^−1^)	0.183	0.090	87	0.186	0.067	97

**Figure 10:**
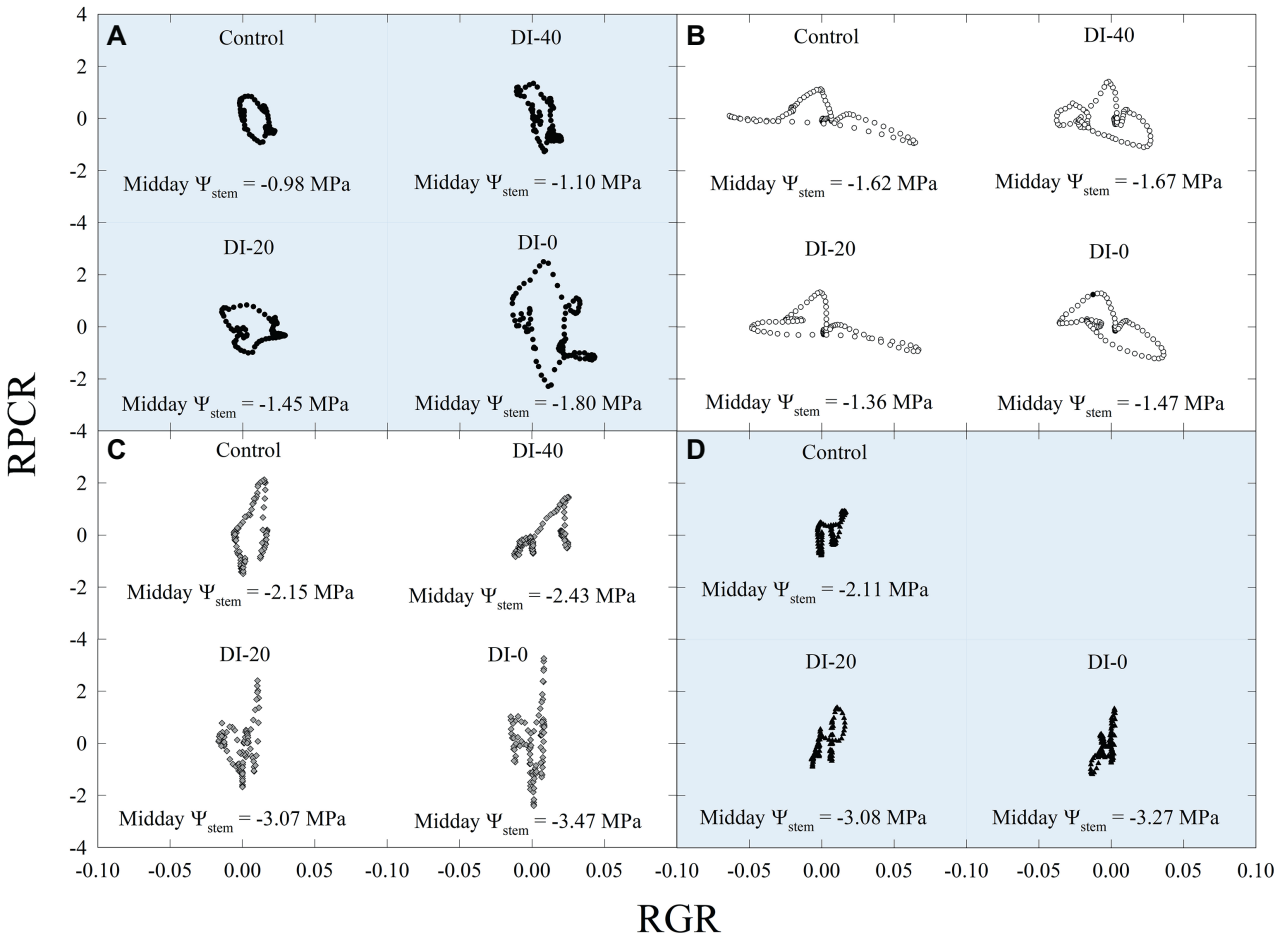
Scatter plots of diel leaf relative pressure change rate (RPCR) and fruit relative growth rate (RGR) at stages I [50 days after full bloom, DAFB **(A)**], II [92 DAFB **(B)**], IIIa [132 DAFB **(C)**], and IIIb [155 DAFB **(D)**] of ‘September Bright’ nectarine fruit growth. Trees irrigated to 100% (control), 40% (DI-40), 20% (DI-20), and 0% (DI-0) of crop evapotranspiration. Midday *Ψ*_stem_ for each irrigation treatment and DAFB is reported in its relative panel.

**Figure 11:**
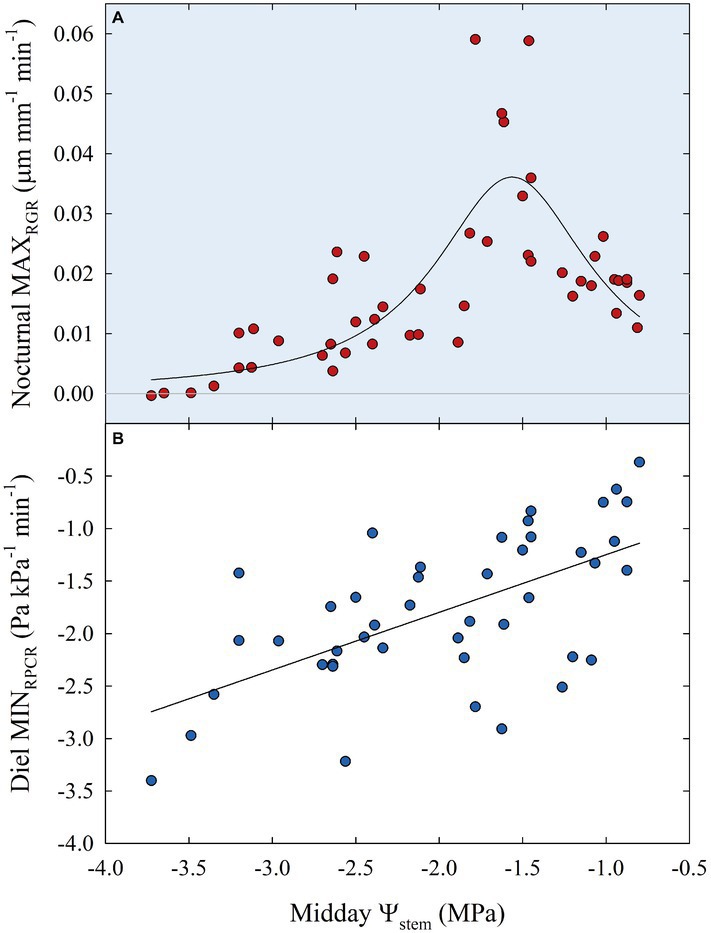
Maximum nocturnal fruit relative growth rate (MAX_RGR_) vs. midday *Ψ*_stem_ **(A)** and minimum diel leaf relative pressure change rate (MIN_RPCR_) vs. midday *Ψ*_stem_ **(B)** for all fruit growth stages and irrigation treatments. Nonlinear regression in panel **(A)**: MAX_RGR_ = 0.04/{1 + [(*Ψ*_stem_ + 1.56)/0.57]^2^}, *R*^2^ = 0.597, *p* < 0.001. Linear regression in panel **(B)**: MIN_RPCR_ = −0.70 + 0.55 × *Ψ*_stem_, *R*^2^ = 0.369, *p* < 0.001.

Subsequently, data of diel relationships (i.e., *p*_p_ vs. FD and RPCR vs. RGR) at 15-min intervals were plotted for a clear sky day at each stage of fruit development. Scatter plots in Figure 10 highlight anticlockwise hysteretic relationships between RPCR and RGR. Similar trends were found for *p*_p_ vs. FD associations (data not shown). Hysteresis among sensors’ outputs and/or water status indicators is common, especially when trunk or leaf indicators are considered (e.g., sap flow density, stomatal conductance, diameter variations, *Ψ*_leaf_, transpiration), and has been widely documented (Brough et al., 1986; Cruiziat et al., 1989; Granier et al., 1989; Ameglio and Cruiziat, 1992; Tognetti et al., 1996; Fernández, 2017). The hysteretic behavior was observed in all the fruit developmental stages, although it showed different patterns (Figure 10). At stage I, there was a gradual increase of the hysteretic loop area as irrigation volume decreased, reaching its maximum size in the DI-0 treatment (Figure 10A). Nevertheless, a similar trend in loop area with higher levels of DI was not observed in the other stages (Figures 10B–D), suggesting stage-dependent mechanisms of water regulation in fruits and leaves. In addition, the generally low midday *Ψ*_stem_ at stage IIIa and IIIb (i.e., ≤ −2.00 MPa) may have altered the hysteretic patterns. Hysteresis is likely to be caused by both a lag in tissue water dehydration and rehydration, and nocturnal/diurnal inverted pattern of the RPCR to RGR association. Consequently, diel RGR and RPCR trends were firstly considered alone and then subdivided into diurnal (7:00 to 19:45 h) and nocturnal (20:00 to 6:45 h) data, to investigate associations with midday *Ψ*_stem_. The use of RGR and RPCR was favored over FD and *p*_p_, as the former yielded the tightest associations with midday *Ψ*_stem_. Diel, diurnal, and nocturnal RGR and RPCR parameters (i.e., RSD, maximum, minimum, sum) from all the irrigation treatments were pooled together and their means were linearly regressed with midday *Ψ*_stem_. Among all the significant (*p* < 0.05) regression models obtained using data from all the stages, the highest *R^2^* were found when nocturnal maximum RGR (MAX_RGR_) (Figure 11A) and minimum diel RPCR (MIN_RPCR_) (Figure 11B) were related to midday *Ψ*_stem_. The nonlinear model in Figure 11A can be explained with the fact that a limited water deficit is needed for maximum fruit cell expansion due to rehydration (i.e., peak at −1.56 MPa). Oppositely, at *Ψ*_stem_ near −1.00 MPa, fruit cell turgor is higher and less water is drawn from nearby organs. When *Ψ*_stem_ reaches particularly low levels (~ −3.50 MPa) maximum RGR tends to zero.

The linear relationship between *Ψ*_stem_ and diel MIN_RPCR_ showed a loose but direct association (Figure 11B), in contrast with findings in olive where Marino et al. (2016), instead, found an inverse linear relationship. In our case, even the linear regression between *p*_p_ (the indicator used by Marino et al., 2016), rather than RPCR, and midday *Ψ*_stem_ resulted in a direct relationship, although with a lower *R^2^* (0.247) than the former (data not shown). The inverse relationship found by Marino et al. (2016) in olive was expected as *p*_p_ is the inverse of turgor pressure, which is instead directly related to *Ψ*_stem_. In our case, diel MIN_RPCR_ indicates the maximum rate over 24 h at which partially dehydrated leaves re-establish some turgor pressure by recalling water from nearby organs. Therefore, the direct relationship between diel MIN_RPCR_ and *Ψ*_stem_ shows that such instantaneous water pulling force increases with water deficit, allowing leaves to maintain minimum hydration and escape desiccation and death. Indeed, a *Ψ*_stem_ < 3.00 MPa could be fatal for nectarine trees if a drought avoidance mechanism is not activated. On the other hand, olive can easily tolerate leaf dehydration at similar levels of *Ψ*_stem_.

Insights from Figures 10, 11 suggested that ratios of RGR to RPCR might be better indicators of midday *Ψ*_stem_, by combining fruit and leaf water relations. More specifically, the changes in hysteretic patterns (Figure 10) indicated that RGR/RPCR variance may be strictly related to midday *Ψ*_stem_ variations, as the shape of the loop changed along with increasing water deficit. However, hystereses were also likely to be influenced by intrinsic parameters of diel, diurnal, and nocturnal variations, such as maximum, minimum, and sum. Consequently, linear regression models considered RGR-to-RPCR ratios for all these parameters regressed vs. midday *Ψ*_stem_. The only two linear models with *R*^2^ > 0.3 were found for nocturnal data using the RSD_RGR_/RSD_RPCR_ (*R*^2^ = 0.346) and MAX_RGR_/MAX_RPCR_ (*R*^2^ = 0.318) ratios. The latter relationship was mostly derived from the significant association found in Figure 11A, as the response to midday *Ψ*_stem_ had a similar peak trend, but with a lower *R*^2^ (0.405). Therefore, the MAX_RGR_/MAX_RPCR_ ratio was discarded.

**Figure 12:**
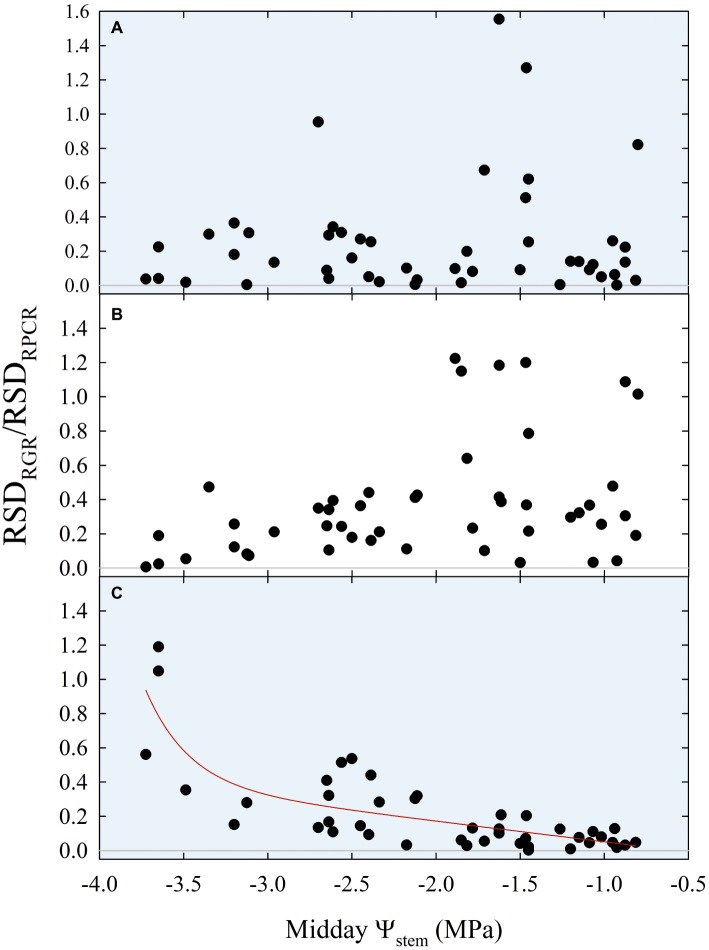
Diel **(A)**, diurnal **(B)**, and nocturnal ratios **(C)** of relative standard deviations of fruit relative growth rate (RSD_RGR_) and leaf relative pressure change rate (RSD_RPCR_) vs. midday *Ψ*_stem_ for all fruit growth stages and irrigation treatments. Expo-linear model in panel **(C)**: RSD_RGR_/RSD_RPCR_ = −0.07 + 2.88E-07 × exp. (−3.89 × *Ψ*_stem_) − 0.12 × *Ψ*_stem_, *R*^2^ = 0.650, *p* < 0.001.

Finally, stepping forward to the strongest association with midday *Ψ*_stem_, the scatter plot showed an inverse nonlinear association (Figure 12C), suggesting that the model might be both composed by a linear phase at higher values of *Ψ*_stem_ and by an exponential phase at lower *Ψ*_stem_. In accordance with our hypothesis, the diurnal regression tended to show an opposite trend, although no significant association was found (Figure 12B). The diel regression reflected the unpredictable hysteretic behavior seen in Figure 10, resulting in the weakest, nonsignificant association (Figure 12A).

The association of nocturnal RSD_RGR_/RSD_RPCR_ to *Ψ*_stem_ (Figure 12C) shifted from linear to exponential at midday *Ψ*_stem_ = −2.3 MPa, suggesting that this water deficit level might be identified as a threshold under which late-ripening ‘September Bright’ nectarine trees are significantly affected by drought. Below the level of −2.3 MPa, the RSD of nocturnal fruit growth increases with respect to the one of leaf turgor pressure. For instance, the slight decrease in fruit diameter occurring between 154 and 161 DAFB in DI-0 trees (Figure 2D) induces an increase in nocturnal RSD_RGR_ while RSD_RPCR_ does not change, generating the observed increase of RSD_RGR_/RSD_RPCR_. At stage III, peach and nectarine stomata become dysfunctional (Chalmers et al., 1983) and high transpiration rates can overcome level of phloem and xylem inflows in fruits (Lescourret et al., 2001; Morandi et al., 2007a). This phenomenon generates particularly low fruit water potential and causes an increase in water potential difference between leaves and fruit (McFadyen et al., 1996), as also found in olive by Fernandes et al. (2018). Therefore, the different regulation of water balance in fruit and leaves may provide a very useful parameter for real-time and continuous monitoring of plant water status.

The identified stage-independent threshold of midday *Ψ*_stem_ (−2.3 MPa) can be used for irrigation management in commercial ‘September Bright’ nectarine orchards under environmental conditions similar to those in our study. However, during stage I, trees exposed to DI (i.e., mean of DI-0, DI-20 and DI-40) never reached such low levels of *Ψ*_stem_, despite yielding fruit with significantly lower final size compared to control irrigated trees (i.e., 53.3 ± 0.44 mm vs. 58.6 ± 0.81 mm for DI and control, respectively). Yet, it is important to acknowledge the limitations of using a significant number of sensors (≥3) on individual fruit and leaves for the appropriate estimation of *Ψ*_stem_ for each tree, and the damage that they may cause when they are kept on the same organ for prolonged time (e.g., limitation of gas exchange, light interception, and growth). In particular, a regular monitoring (at least at weekly intervals) has to be conducted to clamp sensors on different fruit and leaves and to verify their correct use. Even so, the estimated *Ψ*_stem_ from a good sample of trees has the potential to be combined with spatial information (e.g., NDVI and thermal images) for a highly precise irrigation management in modern large orchards.

## Conclusions

Overall, this work highlights a combined fruit and leaf sensing approach for the continuous monitoring of tree water status. On one side, the leaf sensing method guarantees a fast and responsive signal based on leaf turgor pressure that represents a pre-alarm forecast for irrigation management; on the other hand, continuous fruit size sensing provides the precise information on the time-lag and plant dehydration level to which deficit irrigation can be imposed until fruit growth and yield are significantly affected. Both together, leaf and fruit sensing provide a powerful and reliable tool that is not influenced by the fruit development stage and that can be continuously used to detect stem water potential thresholds for irrigation management. In this regard, additional efforts should be made to develop new fruit and leaf sensing technologies that reduce the likelihood to damage organs during the period of data collection. Further investigations need to be carried out to promote models that assess the nocturnal to diurnal shift within the diel hysteresis of fruit growth vs. leaf turgor pressure, and the time lag characterizing the hysteretic loop. Nevertheless, our findings represent an innovative and valid plant-based support to rational and sustainable irrigation management.

## Data Availability

The datasets for this manuscript are available upon request to Agriculture Victoria. Requests to access the datasets should be directed to Mark Glenn O’Connell, mark.oconnell@ecodev.vic.gov.au.

## Author Contributions

All authors contributed to conception and design of the study. MOC, DS, and RLB made available the equipment used in the experiment. AS and MOC carried out field measurements. AS and RLB performed the statistical analysis. AS wrote the first draft of the manuscript. All authors contributed to manuscript revision, read and approved the submitted version.

### Conflict of Interest Statement

The authors declare that the research was conducted in the absence of any commercial or financial relationships that could be construed as a potential conflict of interest.
